# Optical Flow-Based Analysis of the Relationships between Leaf Wilting and Stem Diameter Variations in Tomato Plants

**DOI:** 10.34133/2019/9136298

**Published:** 2019-10-31

**Authors:** Kazumasa Wakamori, Hiroshi Mineno

**Affiliations:** ^1^Graduate School of Integrated Science and Technology, Shizuoka University, 3-5-1 Johoku, Naka-ku, Hamamatsu, Shizuoka 432-8011, Japan; ^2^JST, PRESTO, 4-1-8 Honcho, Kawaguchi, Saitama 332-0012, Japan

## Abstract

The estimation of water stress is critical for the reliable production of high-quality fruits cultivated using the tacit knowledge of expert farmers. Multimodal deep neural network has achieved success in the estimation of stem diameter variations as a water stress index, calculated from leaf wilting and environmental data. However, these studies have not addressed the specific role of leaf wilting in the estimation. Revealing the role of leaf wilting not only ensures the reliability of the estimation model but also provides an opportunity for improving the estimation method. In this paper, we investigated the relationships between leaf wilting and stem diameter variations without resorting to black-box approaches such as deep neural network. To clarify the role of leaf wilting, this study uses cross-correlation analysis to analyze the time lag correlation between leaf wilting, quantified by optical flow, and stem diameter variations as a water stress index. The analysis showed that leaf wilting had a significant time lag correlation with short-term stem diameter variations, which were water stress responses in plants. As the results were consistent with known plant water transport mechanisms, it was suggested that leaf wilting quantified by optical flow can explain short-term stem diameter variations.

## 1. Introduction

Recently, several studies have successfully demonstrated that the use of information technology to monitor key indicators could replace the visual decision-making of expert farmers and prevent the loss of sophisticated cultivation techniques [1–3]. In these studies, data mining, image processing, and machine learning technologies were applied to plant images and environmental data in order to determine and extract indicator variables for such decision-making. Defining an indicator of water stress in cultivation is very important for producing fruits with high sugar content and improving the market value of such products. In Japan, water stress cultivation is commonly used to produce fruits with high sugar content. Such water stress cultivation is currently supported by the tacit knowledge of expert farmers, and a decision-making tool on irrigation control to replace the visual judgement of the expert farmers would be very beneficial. The fruit sugar content ratio can be increased by restricting irrigation and decreasing the amount of water flow into the fruit during stress cultivation, resulting in high sugar content, although the fruit sizes are smaller [4]. However, when plants are exposed to excessive water stress, they may die if they reach permanent wilting point. Therefore, we always need to keep plants under optimal water stress conditions and develop a reasonable method for measuring the water stress condition of plants for optimal decision-making about irrigation.

Several measurement methods have been proposed in previous studies for plant water stress management. Conventionally, water stress changes according to the water potential of a plant. In particular, it depends on the water content of the leaves and the stem. Thus, water stress can be accurately measured based on the water potential of a plant [5]; however, this method cannot be applied to real-time irrigation control systems because it requires the destruction of the plants being measured and hence is unsuitable for time series measurements. Several methods aimed at estimating water stress in real-time irrigation settings have been proposed, which involve estimating various plant-stress responses such as leaf vibration [6] and stem diameter variations [7–16]. The leaf vibration approach measures water stress using an ultrasonic speaker and microphone, while the stem diameter approaches involve employing contact-type displacement sensors. These approaches can be applied to real-time irrigation control systems because water stress is measured with nondestructive sensors in real time. Some characteristics of stem diameter variations in tomato plants have been previously investigated, and it has been noted that the stem diameter has diurnal variations, which are affected by environmental data. In particular, stem diameter decreases with exposure to high solar radiation in the daytime and increases with decreased exposure to solar radiation in the evening [7–9, 12, 15]. In addition, the maximum daily stem shrinkage of stem diameter is related to the vapor pressure deficit [17].

Other water stress estimation methods have been proposed utilizing plant images and environmental data measured without contacting plants [18–26]. Crucially, the contactless measurement allows new farmers to use the water stress estimation method without previous experience of taking such measurements. Contactless approaches make it possible to replace costly sensors such as displacement sensors and ultrasonic speakers that require expert knowledge, with more affordable sensors such as cheap cameras and environmental sensors. By using time series posture images with environmental data, optical flow can be applied to obtain water stress estimates in a densely planted environment—such as with tomato cultivation—to detect the movement of wilting leaves in a complex background [25, 26]. In these approaches, stem diameter variations are estimated as a water stress index using a multimodal deep neural network, which is a machine learning method that uses multimodal data as the input [27]. Plant posture images and environmental data that are used as input data for neural network studies have revealed that the use of multimodal data is effective for estimating stem diameter variations [25, 26]. As previously mentioned, environmental data are known to have a strong influence on stem diameter diurnal variations, and therefore, it is necessary to measure these variations. On the other hand, because these approaches were based on the deep neural network modeling tending to be a black-box process, the role of leaf wilting quantified by optical flow is yet to be clarified and validated. Clarifying what leaf wilting represents can help improve the existing machine learning-based estimation methods for stem diameter variations.

The objective of the current study was to clarify the role of leaf wilting in the estimation of stem diameter variations. Specifically, we aimed to analyze (1) the characteristics of stem diameter variations, which reflect the water stress of plants, (2) the relationships between leaf wilting and stem diameter variations, and (3) the effects of environmental data on the relationships between leaf wilting and stem diameter variations. By linking the results from our analysis to plant water transport mechanisms, we investigated the relationships between leaf wilting quantified by optical flow and stem diameter variations. Finally, we discuss how to improve the performance of machine learning-based estimation methods for water stress using leaf wilting and environmental data.

## 2. Materials and Methods

### 2.1. Data Collection Environment

**Figure 1:**
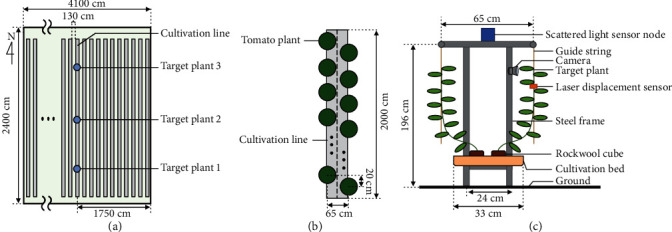
Data collection environment. Overhead view of the greenhouse (a), overhead view of a cultivation line (b), layout of measuring equipment for one targeted tomato plant (c).

We collected plant images, stem diameter, and environmental data from three pinched tomato (*Solanum lycopersicum* L. “Frutica”) plants (labelled target plant 1, target plant 2, and target plant 3) in a dense tomato cultivation from a commercial greenhouse in Fukuroi, Japan (Figure 1). The plants were sown on January 21, 2018, and planted on February 20, 2018; the plants were pinched on April 2, 2018, and crop harvesting began on April 25, 2018. We collected data from April 6, 2018, to April 24, 2018, between pinching and harvesting. For the data collection, we installed an RGB camera (GoPro HERO5 Session, GoPro Inc.), a laser displacement sensor (HL-T1010A, Panasonic Corp.), and a wireless scattered light sensor node [28] for each tomato plant as illustrated in Figure 1(c); the data were collected every minute. The plant image data were collected from 9:00 to 17:00, which was an ideal time range when natural light was sufficiently bright, and other sensor data were collected for the entire day. The RGB cameras were installed at a location where the target tomato was situated at the center of the captured posture images. Laser displacement sensors were located between the 9^th^ and 10^th^ nodes of the tomato plants, and they were connected to a data logger (midi LOGGER GL840, GRAPHTEC Corp.). Wireless scattered light sensor nodes were located above the target plant where the sensor node collected all the environmental data. Each scattered light sensor node had a temperature and humidity sensor (SHT-25, Sensirion) that measured the temperature, relative humidity, and vapor pressure deficit (VPD) and silicon photodiodes (S1133-14, Hamamatsu Photonics K.K.) that measured scattered light. The sensor node was shaped like a cube covered with vinyl chloride plates except for one surface, for sunlight protection; the two sensors were attached to the inside of the box. Since the measured scattered light inside the box was not affected by the shadows of steel pipes in the greenhouse, the value always represented the overall brightness around the target plant [29]. In addition, scattered light data were converted from lux to photosynthetic photon flux density (PPFD) (*μ*mol · s^−1^m^−2^) by dividing the value by a conversion factor of 54 [30].

### 2.2. Cultivation Method and Growth Environment

In the present study, we designed a new growing system for tomato plants with high density planting and low-truss cultivation to collect time series cultivation data. This system used a rockwool culture (Yasaihana-pod, Nippon Rockwool Corp.) for each tomato plant, and the plant density was 4 plants/m^2^. Since the rockwool culture was shaped like a small cube (6cm × 6cm × 6cm), the roots of the plant were restricted in the cube. The system irrigated the culture with a nutrient solution (electrical conductivity 2.2 dS/m, pH 6.7). The irrigation control was based on their measured stem diameter and an indexed “relative stem diameter calculated from the most recent irrigation (RSR).” The rsr_*t*_ from which the RSR value in current time (*t*) is calculated was as follows:

(1) $$\begin{array}{c} \text{rs}{\text{r}}_{t}=\frac{\text{ste}{\text{m}}_{t}}{\max\left(\text{ste}{\text{m}}_{t-n},\text{ste}{\text{m}}_{t-n+1},\cdots ,\text{ste}{\text{m}}_{t}\right)}, \end{array}$$

where *t* means the current time and *n* means the elapsed time since the last irrigation; thus, RSR is a value calculated by dividing the current stem diameter by the maximum stem diameter achieved at the most recent irrigation event. We set a threshold *T*_rsr_ for RSR, and when the RSR fell below the threshold, an irrigation system irrigated the rockwool cube. After irrigation, the stem diameter gradually increased by absorbing water and the RSR value was set to 1.0000. When the stem diameter decreased because of water stress, the RSR fell below 1.0000. Therefore, when the threshold *T*_rsr_ was set to less than 1.0000, the irrigation control caused water stress to the plants. The present study required a sufficient irrigation interval to cause water stress to the plants because the irrigation impact on the water status was not instantaneous. The RSR-based irrigation control helped adjust the optimal interval based on plant water stress using the threshold *T*_rsr_. We defined the threshold *T*_rsr_ based on the opinions of experienced farmers in this experiment. *T*_rsr_ was 0.9950 from April 6, 2018, to April 7, 2018, and 0.9975 from the April 8, 2018, to April 24, 2018. The automatic irrigation control worked from sunrise to 19:00 each day. Sunrise was defined as when the PPFD reached 14.8*μ*mol · s^−1^m^−2^ and each irrigation event was 15 ml of water per plant.

### 2.3. Data Preprocessing

**Figure 2:**
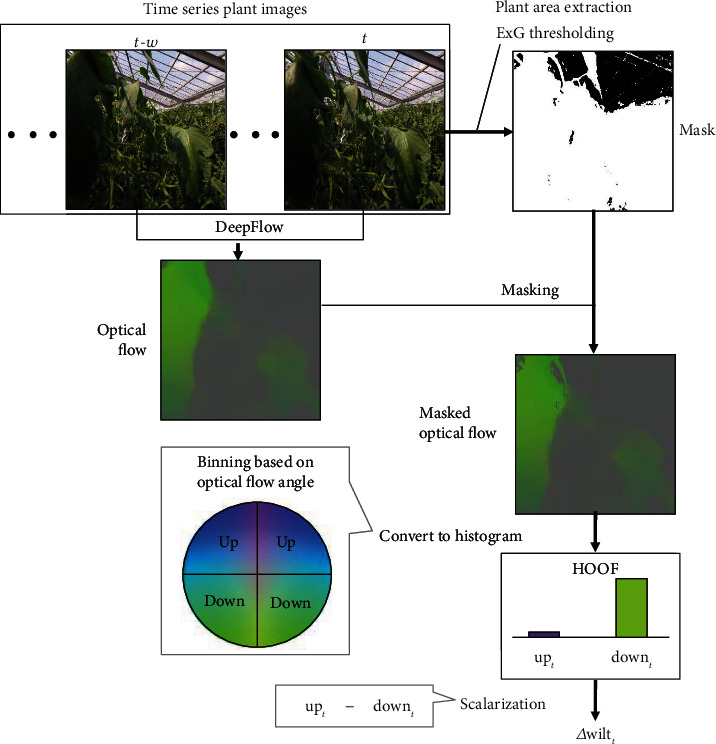
Processing flow of leaf wilting quantification from time series plant images. The parameter *w* indicates sampling periods used in calculating the optical flow. Note that we set *w* = 5min for DeepFlow to sufficiently detect leaf wilting. Time series plant images were resized to 144 × 144 pixels in each image.

Plant stress responses including leaf wilting and stem diameter were affected by diurnal environmental variations [15]. However, diurnal variations were not controlled by irrigation, which was able to control only short-term variations in stress responses. To analyze the relationships between leaf wilting and stem diameter variations considering irrigation, this study compared only short-term variations of leaf wilting and stem diameter. Thus, this analysis focused on the short-term variations of leaf wilting and stem diameter and excluded diurnal variations. To extract information about short-term leaf wilting and stem diameter variations, we used optical flow of plant images and temporal variations in stem diameter. Optical flow is an image processing method that calculates pixel-level velocity vectors, which are composed of angle and magnitude, between two images. In other words, optical flow expresses the relative motion pattern of objects in the two images. In this study, we quantified relative leaf wilting using the optical flow from the time series plant posture images.  Figure 2 shows the workflow of image processing for the quantification of relative leaf wilting. The image processing quantified relative leaf wilting (Δwilt_*t*_) at time *t* and *t* − *w*. Firstly, an optical flow algorithm detected the wilting motion of leaves. In this study, we used DeepFlow [31], which is one optical flow algorithm that detects the relative motion of nongrid objects, such as wilting at high density and robustly from a complex background. Secondly, an excess green (ExG) index is calculated for masking the detected optical flow because optical flow detection involves the objects in motion such as clouds from the sky in the background. We extracted the plant area in the plant images with a color filter (threshold of 0.0) based on the ExG index to generate masked optical flow. Thirdly, we converted the masked optical flows to a histogram called the histograms of oriented optical flow (HOOF) [32]. HOOF shows each optical flow vector according to its angle and is weighted on its magnitude. We have a hypothesis that downward angles indicate leaf wilting, whereas upward angles depicted a recovery of wilting in optical flow from the time series plant images. Thus, we generated an elementary histogram based on the upward bin (up_*t*_) and downward bin (down_*t*_) to enable the analysis program to reveal the features that related to leaf wilting. Finally, we calculated a scalar value (Δwilt_*t*_) that represented relative wilting by subtracting down_*t*_ from up_*t*_.

For time series stem diameters (stem = {stem_1_, stem_2_, ⋯, stem_*n*_}), we calculated temporal differences in stem diameter (Δstem_*t*_) as follows:

(2) $$\begin{array}{c} \Delta {\text{stem}}_{t}={\text{stem}}_{t}-\text{ste}{\text{m}}_{t-w}, \end{array}$$

where Δstem_*t*_ indicates short-term variations in the stem diameter and the relative value between time *t* and *t* − *w* as with Δwilt_*t*_. By calculating the temporal differences, we can remove the diurnal variations of stem diameter and avoid spurious correlations caused by diurnal variations in the data analysis. Therefore, we can obtain an estimation of the relationships between leaf wilting and short-term stem diameter variations.

In this analysis, we used *w* = 5min, which is half of the minimum irrigation interval (10 min), for calculating the optical flow of time series plant images and temporal differences in stem diameter. Choosing the interval *w* involved a trade-off between removing diurnal variations in stem diameter and leaf wilting quantification. A shorter *w* could remove diurnal variations in stem diameter clearly; however, it would not be able to detect slight leaf wilting by optical flow. On the other hand, a longer *w* could detect the slight leaf wilting, but the diurnal variations remained in Δstem. Moreover, this study set the interval *w* to be less than the minimum irrigation interval. If *w* was above the minimum irrigation interval, two or more irrigations could be included in the interval *w*, in which case, we could not have analyzed the short-term relationships affected by the irrigation. In addition, Δwilt and Δstem were calculated at 1 min intervals of a moving window.

### 2.4. Data Analysis

We analyzed the relationship between Δwilt and Δstem using cross-correlation analysis that could evaluate the correlation and time lag. It was assumed that Δwilt and Δstem have temporal dependencies caused by water transport in a plant, and thus, the cross-correlation analysis can find temporal dependence. In this analysis, we slid Δstem along the time series, and at every lag position *l*, we calculated the correlation coefficient with Δwilt. The correlation was expressed by the cross-correlation coefficient *C*(*l*):

(3) Cl=ΔstemilΔwilti−μΔstemμΔwiltσΔstemσΔwilt,

where 〈_*l*_Δstem_*i*_Δwilt_*i*_〉 is the expected value of _*l*_Δstem_*i*_Δwilt_*i*_ for all possible values of *i*. *μ*_Δstem_ and *μ*_Δwilt_ are the average values, and *σ*_Δstem_ and *σ*_Δwilt_ are the standard deviations of Δstem and Δwilt, respectively. We used the “numpy.correlate” function in Python 2.7 to calculate ∑_*l*_Δstem_*i*_Δwilt_*i*_, and we divided the return values by *n* − ∣*l*∣ to calculate the expected value. In this study, we calculated the cross-correlation coefficient for each day separately, because the cross-correlation coefficient was not applicable to time series data with missing information such as the lack of image data at night. Moreover, as the greenhouse environmental conditions differed with daily weather, it was possible to examine whether the relationships between leaf wilting and stem diameter variations were dependent on cross-correlation coefficient calculations of environmental data for each day. In the cross-correlation coefficient, the sign of *l*_max_ means time lag at the highest correlation coefficient and represents the direction of the temporal dependence relationships between the two time series data. A positive *l*_max_ means that the stem diameter variations preceded leaf wilting, while a negative *l*_max_ indicates the opposite temporal relationship.

Then, we analyzed the relationships between the maximum cross-correlation coefficient and the mean of environmental data to clarify the environmental condition in which leaf wilting had the closest relationships to the stem diameter variations. The mean of the environmental data is an arithmetic mean and was calculated from data collected from 9:00 to 19:00; the time range was based on the time of plant image collection. Linear, second-order polynomial, piecewise, log, and exponential regressions were used for regression analysis between the maximum cross-correlation coefficient and environmental data. The piecewise regression split the explanatory variables and made two regression lines. The threshold for splitting the regression line was fitted as a parameter in the regression method. The regressions were fitted using the “scipy.optimize.curve_fit” function in Python 2.7. The *R*^2^ score was applied to select the most probable regression model.

## 3. Results

### 3.1. Diurnal Variations and Short-Term Variations in Stem Diameter

**Figure 3:**
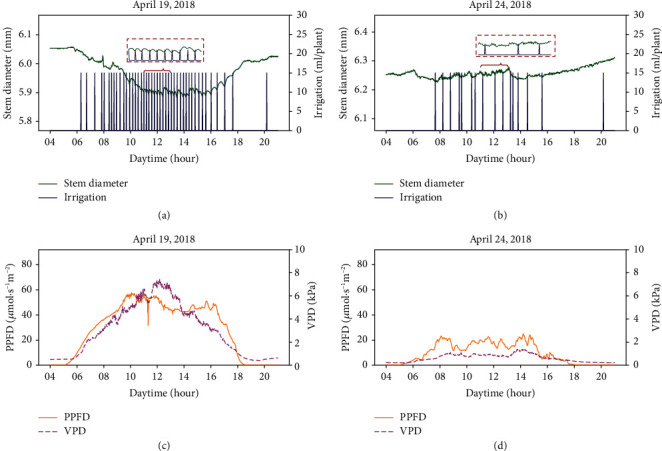
Typical variations in stem diameter. Diurnal variations and short-term variations in stem diameter and irrigation on a sunny day (a) and on a cloudy day (b). PPFD and vapor pressure deficits in the two days (c, d). All data were collected from target plant 2, and daily averages of the PPFD were maximum and minimum on April 19, 2018, and April 24, 2018, respectively.

To examine the characteristics of stem diameter variations, the stem diameters of tomato plants were measured over time using laser displacement sensors.  Figure 3 shows the stem diameter variations on a sunny day and a cloudy day. The stem diameter on a sunny day exhibited larger diurnal variation than that on a cloudy day. On a sunny day, the stem diameter decreased after sunrise and increased after sunset, and the diurnal variation was related to scattered light intensity. We confirmed that the characteristic diurnal variations were in agreement with the results of previous studies [7–9, 12, 15]. However, stem diameter included not only diurnal variations but also short-term variations (red dotted line area in Figures 3(a) and 3(b)). Because short-term variations are repeated changes based on irrigation and the variations are notable on a sunny day, it was assumed that short-term variations in stem diameter were related to water uptake. To analyze irrigation-based relationships between leaf wilting and stem diameter, the short-term variations of leaf wilting and stem diameter were compared.

### 3.2. Relationships between Relative Leaf Wilting and Short-Term Stem Diameter Variations

**Figure 4:**
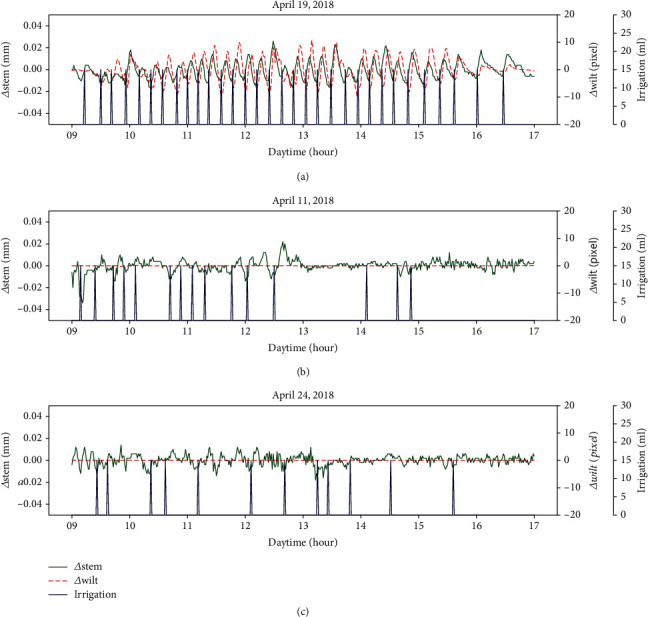
Examples of relative leaf wilting (Δwilt) and temporal differences in stem diameter (Δstem). An example on a day which leaf wilting was detected (a), examples on days which leaf wilting was not detected (b, c) in target plant 2.

We subsequently analyzed the relationship between Δwilt and Δstem using cross-correlation analyses.  Figure 4(a) shows Δwilt and Δstem data from a day where leaf wilting was detected, and Figures 4(b) and 4(c) show the same data from days where leaf wilting was not detected. Δstem was time series data, where diurnal variations were removed by adjusting for temporal differences to the stem diameter; the units are in mm. Δwilt was time series data calculated from the optical flow of the time series plant images in the same intervals as Δstem. Δwilt indicates an average value per pixel of the optical flow vectors, and the units are in pixels. Both Δstem and Δwilt represent the amount of relative displacement in a 5 min interval.

**Figure 5:**
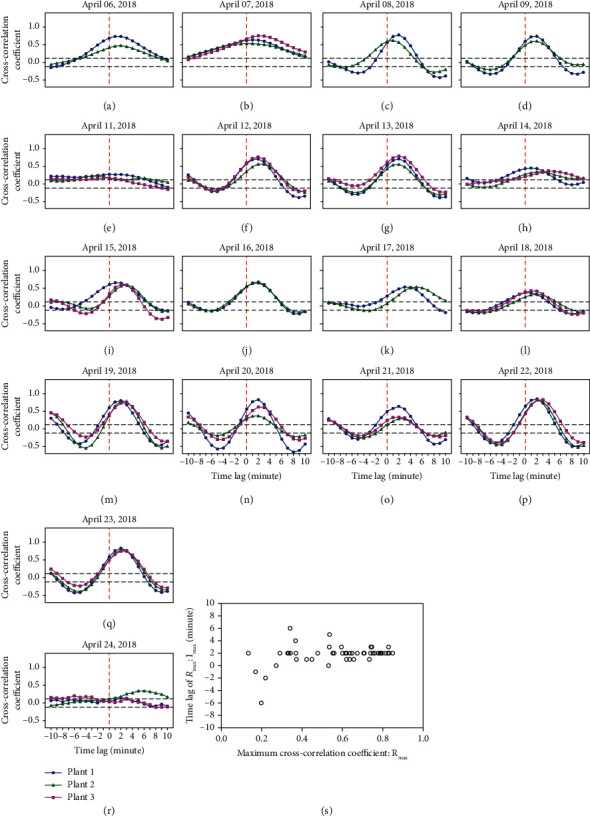
Cross-correlation coefficients. Cross-correlation graphs between Δwilt and Δstem from 9:00 to 17:00 for each day (a–r). The red dotted line shows lag = 0 and the two black dotted lines represent the 99% confidence interval. A graph on April 10, 2018, and target plant 3's lines on (a), (c), (d), (j), and (k) are not shown because of missing plant images on those days. A scatter plot represents the relationships between the maximum cross-correlation coefficients and the time lags (s).

Figures 5(a)–5(r) show cross-correlation graphs of Δstem and Δwilt. The *x*-axis of the figures indicates a delay of Δstem in the cross-correlation analysis and the unit of the time lag is minutes. A time lag of 0 means no time lag, less than 0 means delayed Δstem for negative temporal direction, and greater than 0 means delayed Δstem for positive temporal direction. We confirmed significant positive correlations between Δstem and Δwilt from these graphs as shown in Figures 5(a)–5(d) and 5(f)–5(q). The results show that Δwilt and Δstem have a positive correlation with a time delay. *R*_max_ was the maximum cross-correlation coefficients confirmed at a time lag of 0 or more during this time; thus, it is suggested that Δwilt had a slight time delay compared to Δstem.

Figure 4(a) shows a time series plot of Δwilt and Δstem on a sunny day when *R*_max_ = 0.789 and *l*_max_ = 2 were recorded. Both Δwilt and Δstem increased from the irrigation time points and then both responses decreased. In addition, it appears that Δwilt is delayed when compared to Δstem. On the other 2 days, the cross-correlation graphs do not change periodically (Figures 5(e) and 5(r)), because leaf wilting did not occur, and Δwilt remained nearly unchanged as shown in Figures 4(b) and 4(c). Moreover, the weather on the two days was mostly cloudy.

Figure 5(s) presents a scatter plot of *R*_max_ and *l*_max_, time lags of *R*_max_. When *R*_max_ is high, the time lag converges at 2 min; the average of *l*_max_ is 1.96 min when *R*_max_ > 0.6 (the maximum of *R*_max_ is 0.849 on April 22, 2018). Thus, this indicates that leaf wilting or recovery occurs approximately 2 min after the stem diameter changes in this environment. Leaf wilting occurs noticeably on sunny days because of water stress.

### 3.3. Relationship between Maximum Cross-Correlation Coefficient and Environmental Data

**Figure 6:**
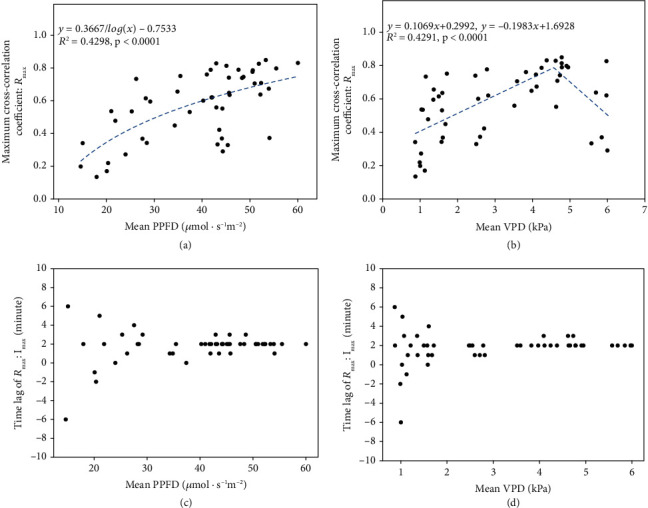
Relationships between cross-correlation and environmental data. Relationships between the maximum cross-correlation coefficient and the mean PPFD (a) and mean VPD (b). The relationships between the time lags and mean PPFD (c) and mean VPD (d).

We finally analyzed the relationship between environmental data and the maximum cross-correlation coefficient of Δwilt and Δstem or its time lag to confirm that the correlation strength was affected by the greenhouse environmental conditions. The maximum cross-correlation coefficient between the two stress responses represented the strength of the linear relationship in terms of the time delay between the two time series data. Figures 6(a) and 6(b) show a scatter plot of the maximum cross-correlation coefficients and mean environmental data. The figures show that the maximum cross-correlation coefficient is related to the mean PPFD and mean VPD suggesting that the maximum cross-correlation between the Δwilt and Δstem coefficients was affected by environmental data. The *R*^2^ scores with the mean PPFD and VPD were 0.4298 and 0.4291, respectively. The maximum cross-correlation coefficient increased logarithmically with increase in the mean PPFD as shown in Figure 6(a) (*p* < 0.0001), and the relationship with mean VPD was best fit with a piecewise linear function as shown in Figure 6(b) (*p* < 0.0001). In addition, the time lag between Δwilt and Δstem converged at about 2 min with an increasing mean PPFD or mean VPD as shown in Figures 6(c) and 6(d). When the mean PPFD exceeded 40*μ*mol · s^−1^m^−2^, the average time lag was 1.96 min between Δwilt and Δstem and the maximum and minimum time lags were ±1 min from 2 min. In the case of VPD exceeding 2 kPa, the average time lag was 2.00 min and the maximum and minimum time lags were ±1 min from 2 min, likewise the mean PPFD.

## 4. Discussion

We studied the relationships between leaf wilting quantified by optical flow and stem diameter variations with the objective of clarifying the role of leaf wilting in the estimation of stem diameter variations. We analyzed the relationships between relative leaf wilting time and short-term stem diameter variations using cross-correlation analyses. Image processing algorithms, including optical flow, aided calculation of relative leaf wilting values, and temporal differences in stem diameter variations were measured. The results showed that (1) the short-term variations were correlated with leaf wilting and the two data had a time lag and (2) the strength of the correlation between leaf wilting and short-term stem diameter variations was affected by environmental data. Finally, the observed results were supported by plant water transport literature.

### 4.1. Water Transport-Based Interpretation of Results

Water transportation in a plant is caused by transpiration during the day [33]. When osmotic pressure, which is involved in the water potential, is decreased and negative pressure is exerted in the leaves by transpiration, the water potential gradient transports water from the roots and stems to the leaves. If water is available from the irrigated soil at this time, the root will have a positive pressure and water can continue to be transported to the leaves. If water is not available from the soil, the water potential is reduced, the stem diameter is reduced, and the plant becomes depleted of water and the leaves wilt. The water potentials of stem and leaves successively change through water osmosis from the root. The above sequence might indicate that the stem is water stressed, which manifests as stem diameter variations, and precedes the leaf water potential change, which appears as wilting. In this study, we investigated the time lag correlation between the two plant responses (Figure 5), and we found a significant correlation. In the scatter plot of time lag and maximum correlation coefficient (Figure 5(s)), the average time lag was 1.96 min, when the maximum correlation coefficient was 0.6. Thus, the short-term stem diameter variations preceded leaf wilting by approximately 2 min, and this result supported the hypothesis that the relationships between the water potential variations in the stems and leaves correlated with each other in the short-term and the relationship has a time lag. Therefore, it was concluded that the time required for the transport of water from the stems to the leaves was 2 min according to this analysis.

When the transpiration rate was correlated with the relative leaf wilting and short-term stem diameter variations, the strength of their relationship was affected by environmental data, which affected the transpiration rate. We analyzed the relationship between mean of environmental data (PPFD and VPD) and the maximum cross-correlation coefficient. The maximum cross-correlation coefficient indicated the strength of the relationships between relative leaf wilting and the short-term stem diameter variations (Figures 6(a) and 6(b)). These analyses showed that the maximum correlation coefficient was related to these environmental data. In particular, the maximum correlation increased with increasing PPFD, indicating solar radiation or increasing VPD of 0–4 kPa. The characteristics were similar to relationships between these environmental data and sap flow or transpiration [34, 35]. The maximum correlations with the VPD did not increase from 4 kPa, and therefore, it is conjectured that stomatal closure caused by high VPDs inhibited transpiration and leaf wilting. Therefore, the results suggested that the strength of the relationships between relative leaf wilting and stem diameter variations may be affected by environmental data. In the same way, environmental data affected the time lag of the relationship. It appeared that the time lag converged when the mean PPFD and VPD values increased (Figures 6(c) and 6(d)). In addition, the average time lag was around 2 min in conditions where mean VPD > 2kPa and mean PPFD > 40*μ*mol · s^−1^m^−2^ in this environment. These results suggested that stem diameter variations preceded leaf wilting by approximately 2 min in environmental conditions where leaf transpiration is facilitated.

Furthermore, the analysis of these results showed that relative leaf wilting, quantified by optical flow, and short-term variations of stem diameter had time lag correlations with environmental conditions where transpiration was constantly promoted. These results could be explained by the water transport mechanism. Thus, it is concluded that the optical flow-based leaf wilting can explain short-term variations of stem diameter caused by transpiration during the day.

### 4.2. Improvement of Stem Diameter Variation Estimation by Multimodal Machine Learning

Since our results suggested that the correlation between Δwilt and Δstem occurred only under conditions when the transpiration rate was high, only using optical flow-based monitoring of water stress may not be useful for irrigation under other environmental conditions. Alternatively, modeling based on multimodal machine learning can estimate stem diameter variations irrespective of whether the environment facilitates transpiration. The multimodal machine learning method can use leaf wilting data and environmental data dynamically to estimate stem diameter variations which are then used as a water stress index. Thus, when leaf wilting occurred, the model estimated stem diameter from both leaf wilting and environmental data, and when leaf wilting did not occur, the model estimated only using the environmental data. The modeling by multimodal machine learning achieved the stem diameter-based irrigation in a nondestructive manner from leaf wilting and environmental data.

In this study, we quantified the relationships between relative leaf wilting and short-term stem diameter variations using optical flow and cross-correlation analyses. Moreover, we noted how the strength of the relationships between these parameters was associated with environmental data. These results verified the effectiveness of using time series plant posture images and environmental data as input data in existing estimation methods of stem diameter variations [25, 26]. Several studies have reported that environmental data affect the diurnal variation of the stem diameter [15]; thus, the existing methods can estimate such diurnal variation using environmental data. In addition, the existing methods could estimate this short-term variation by superposing features of leaf wilting extracted from plant images and environmental data which were related to short-term stem diameter variations. Therefore, we assumed that existing methods could estimate stem diameter variations because they used two types of input data that characterize the diurnal and short-term variations in stem diameter. Moreover, the analysis provided an approach that improved stem diameter estimation of plant images and environmental data using a deep neural network. Recurrent neural network layers, such as long short-term memory (LSTM) [36] and gated recurrent unit (GRU) [37], were able to extract time series leaf wilting features after considering time lags with short-term stem diameter variations, as with the existing method [25]. However, different temporal features should be extracted from plant posture images and environmental data in deep neural networks, because the two-type input data have different temporal relationships with stem diameter variations. Environmental data and leaf wilting explained diurnal and short-term variations of stem diameter, respectively. Thus, a neural network with one general LSTM layer, as in the existing method [25], seemed inadequate to estimate stem diameter variations. We expected that recurrent neural networks that can extract multiple levels of temporal features such as stacked LSTM [38], tensorized LSTM [39], and nested LSTM [40] would all be suitable for estimating stem diameter variations, and we should investigate the improvement in deep neural networks for water stress estimation in future work.

### 4.3. Limitations and Future Work

Our results had four limitations. First, other environmental conditions may not cause the relationships between leaf wilting and stem diameter variations that we described here. In the analysis, we assumed equilibrium conditions of transpiration, which were based on the fact that environmental conditions did not change within intervals of *w* = 5min. Therefore, the time lag correlation was not observed in the transpiration disequilibrium conditions such as on a cloudy day. In transpiration disequilibrium conditions, the stem diameter variations can affect leaf wilting status based on changes in the driving force of transpiration. Future studies are needed to develop an analysis method that can elucidate the relationship between the stem diameter and leaf wilting under various environmental conditions including transpiration disequilibrium.

Second, we analyzed tomato plants after patching; the transpiration rate and maximum water potential are different in each type of plant, and they are also affected by its growth stage. Thus, the quantified results including the correlation coefficient, time lag, and the regression equation applied to tomato plant may not be generalizable to other plants. On the other hand, many studies have reported that stem diameter variations are expressed as a result of water stress in various types of plants [7, 9, 11, 13, 14], and these plants generally exhibited leaf wilting caused by an imbalance between the water uptake rate and transpiration rate. Thus, it is expected that the relationships between leaf wilting and variations of the stem diameter could be elucidated for various types of plants by applying our analysis methods.

Third, we analyzed only the relationship between relative leaf wilting and relative stem diameter in response to water stress in this study. There are many biological factors related to plant water stress or transpiration, such as soil water potential, stomatal conductance, and CO_2_ levels, and it is unclear if there are relationships between our results and these factors. An analysis of the relationships of stem diameter variations with these biological factors may reduce the residual from the results of the maximum cross-correlation coefficient and PPFD or VPD (Figures 6(a) and 6(b)). However, it was a limitation of this study, and in the future, we should collect data related to these biological indices and conduct an exhaustive analysis. In addition, the results were based on only one set of cultivation conditions and the environmental data were not independent; thus, these thresholds of PPFD and VPD might be dependent on these cultivation conditions. In a future study, we should analyze data from cultivations under various stresses to evaluate the generality of these environmental thresholds.

Finally, a plant experiences both leaf rolling and leaf wilting with limited irrigation [41]. The optical flow-based method cannot detect and quantify leaf rolling; however, it is necessary to do so to develop a more robust irrigation control mechanism based on the total leaf condition. Leaf rolling leads to a change in its three-dimensional (3D) shape, and it is possible that this can be detected with a depth camera that can measure both an RGB image and depth data in 3D. We would like to examine the applicability of the depth camera for leaf condition quantification in future studies.

## Supplementary Materials

Supplementary Materials Supplementary Fig. 1: box-and-whisker plot of daily environmental data in the growth environment. Each box-and-whisker includes environmental data from 9 am to 7 pm, as shown in Figure 5. The maximum and minimum of whisker represent the maximum and minimum of each environmental data, respectively.
