# Supplementary figures and images for: Optical Flow-Based Analysis of the Relationships between Leaf Wilting and Stem Diameter Variations in Tomato Plants
Source: Plant Phenomics. 2019 Oct 31;2019:9136298. doi: 10.34133/2019/9136298 (PMC7706306; doi:10.34133/2019/9136298)

## Slide 1
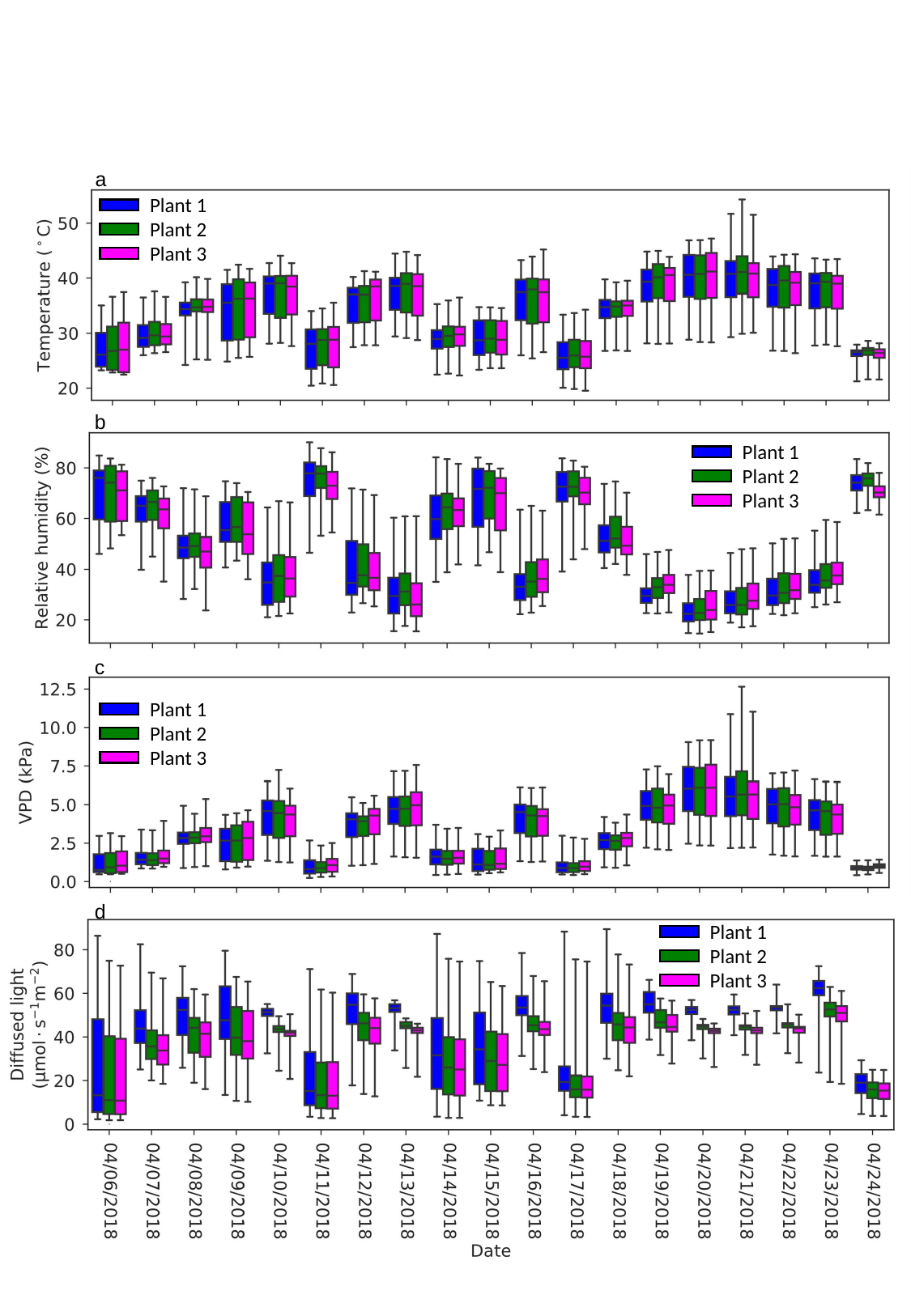

a
Plant 1
Plant 2
Plant 3
b
Plant 1
Plant 2
Plant 3
c
Plant 1
Plant 2
Plant 3
d
Plant 1
Plant 2
Plant 3

Supplement: Supplementary Materials — Supplementary Fig. 1: box-and-whisker plot of daily environmental data in the growth environment. Each box-and-whisker includes environmental data from 9 am to 7 pm, as shown in Figure 5. The maximum and minimum of whisker represent the maximum and minimum of each environmental data, respectively. [file 9136298.f1.pptx]
